# Aiming for the complete utilization of sugar-beet pulp: Examination of the effects of mild acid and hydrothermal pretreatment followed by enzymatic digestion

**DOI:** 10.1186/1754-6834-4-14

**Published:** 2011-05-31

**Authors:** Stefan Kühnel, Henk A Schols, Harry Gruppen

**Affiliations:** 1 Laboratory of Food Chemistry, Wageningen University, Bomenweg 2, 6703 HD Wageningen, the Netherlands

## Abstract

**Background:**

Biomass use for the production of bioethanol or platform chemicals requires efficient breakdown of biomass to fermentable monosaccharides. Lignocellulosic feedstocks often require physicochemical pretreatment before enzymatic hydrolysis can begin. The optimal pretreatment can be different for different feedstocks, and should not lead to biomass destruction or formation of toxic products.

**Methods:**

We examined the influence of six mild sulfuric acid or water pretreatments at different temperatures on the enzymatic degradability of sugar-beet pulp (SBP).

**Results:**

We found that optimal pretreatment at 140°C of 15 minutes in water was able to solubilize 60% w/w of the total carbohydrates present, mainly pectins. More severe treatments led to the destruction of the solubilized sugars, and the subsequent production of the sugar-degradation products furfural, hydroxymethylfurfural, acetic acid and formic acid. The pretreated samples were successfully degraded enzymatically with an experimental cellulase preparation.

**Conclusions:**

In this study, we found that pretreatment of SBP greatly facilitated the subsequent enzymatic degradation within economically feasible time ranges and enzyme levels. In addition, pretreatment of SBP can be useful to fractionate functional ingredients such as arabinans and pectins from cellulose. We found that the optimal combined severity factor to enhance the enzymatic degradation of SBP was between log R'_0 _= -2.0 and log R'_0 _= -1.5. The optimal pretreatment and enzyme treatment solubilized up to 80% of all sugars present in the SBP, including ≥90% of the cellulose.

## Background

Sugar-beet pulp (SBP) consists of up to 75% w/w of carbohydrates (dry matter). Arabinose, glucose and galacturonic acid (GA) are the main sugar moieties present in complex polysaccharide structures [1]. After sugar extraction, the pressed pulp has a dry-matter content of 18-23% w/w. To date, SBP has been used mainly as animal feed or, in regions with no livestock farming, dumped in landfill [2,3]. The low dry-matter content makes combustion of SBP for heat and power production unfavorable; however, its low lignin and high sugar content make it an interesting candidate for biorefinery and/or bioethanol production. The availability of new, pentose-fermenting yeast strains allows the efficient use of biomass for bioethanol production [4]; however, no yeast strains that allow the fermentation of uronic acids to ethanol are currently available.

For fermentation, the cell-wall material needs to be degraded into fermentable monosaccharides. To this end, lignocellulosic feedstocks are often structurally modified by a mild pretreatment before enzymatic release of fermentable monosaccharides [5]. The severity of this pretreatment can be measured by a combined severity factor (log R″_0_) that takes into account pretreatment time, pretreatment temperature, and acid concentration (w/w%) and pH after pretreatment [6,7,47]. In general, a pretreatment has to be strong enough to disrupt the cellulose-hemicellulose network and, if present, the cellulose-lignin network; however, as the severity of the pretreatment increases, more biomass is degraded to non-fermentable products and products toxic to yeast, such as furfural or hydroxymethylfurfural (HMF) [8]. Along with release of furfural and HMF, weak acids (such as acetic acid) which can have a negative effect on yeast growth, viability and fermentation, may also be released [9,10]. Although yeasts can adapt to moderate levels of furfural and detoxification, as shown previously [11], it is economically and ecologically favorable to avoid the formation of such compounds.

During the past decades, much work has been performed on the degradation of SBP, which has shown good yields of solubilized carbohydrates; however, in these studies, high levels of enzymes or chemicals were required [12-15]. To our knowledge, there are no data available for the degradation of SBP in commercially reasonable time spans and with economically feasible enzyme levels.

In this study, we examined the effect of hydrothermal or mild acid pretreatment on enhancing the enzymatic degradation of SBP. Several different pretreatments were used, with different temperatures and pH values. The pretreated samples were characterized and used for an enzymatic saccharification study to analyze the amounts of fermentable monosaccharides produced.

## Methods

### Starting material

SBP was obtained as frozen pressed pulp (23% dry-matter content, harvest 2006; Suiker Unie, Dinteloord, The Netherlands). The pulp contained 68% w/w carbohydrates (dry weight). The constituent sugars were arabinose (18% w/w), glucose (22% w/w), uronic acids (UAs) (18% w/w), galactose (5% w/w), rhamnose (2% w/w), xylose (2% w/w) and mannose (1% w/w), respectively, and the pulp contained 4% w/w residual saccharose. Other components were protein (8% w/w), lipids and salts. Ferulic acid (0.5% w/w), acetic acid (1.6% w/w) and methanol (0.4% w/w) were present as ester-linked substituents of polysaccharides.

### Pretreatment

Pretreatments were carried out with a dry-matter content of 5% w/w: 152 g of pressed pulp was made up to 700 g with distilled water. The pulp was mixed for 5 minutes using a blender (type 7011G; Waring Commercial, Torrington, CT, USA) at low speed. Concentrated sulfuric acid (180 μl) was added to the blended pulp, according to the conditions used (Table 1). Pretreatment was performed in a pressure reactor (Model 4520; Parr Instrument Company, Moline, IL, USA) connected to a power controller (Model 4875; Parr Instrument Company) and operated by a process controller (Model 4875; Parr Instrument Company). The reactor was equipped with an oil bath (200°C) that caused oil to flush through a spindle inside the reactor. An electric heating cord was attached to the outside of the reactor to accelerate the average heating rate and to maintain a constant temperature after heating. The oil bath was turned off when a temperature 20°C below the desired temperature was reached in the reactor. After 12 minutes, the electric heater was removed, and the reactor was cooled by flushing tap water (at 10°C) in a specially designed flow-through water bath, resulting in an overall pretreatment time of 15 minutes at the pretreatment temperature chosen. Temperature and pressure were recorded, and the pH of the samples was determined before and after the pretreatment. Directly after the pretreatment, 1 ml of supernatant was removed and filtered through a 0.2 μm round filter (Whatman plc, Maidstone, Kent, UK) to determine the free amounts of lactic acid, acetic acid, formic acid, furfural and hydroxymethyl furfural. The severities of the pretreatments were calculated as the combined severity factor [6,7,47]:

**Table 1:** Pretreatment conditions of sugar-beet pulp

Sample	Temp, °C	**H**_**2**_**SO**_**4**_^**a**^	**pH**^**b**^	**R″**_**0**_^**c**^	**log R″**_**0**_^**d**^
0-0	0	0	5.3	-1.77	-6.97
120-0	120	0	4.8	1.76	-3.04
140-0	140	0	4.4	2.35	-2.05
170-0	170	0	4.1	3.24	-0.86
120-1	120	1*	3.9	2.11	-2.14
140-1	140	1*	3.9	2.70	-1.65
170-1	170	1*	3.8	3.62	-0.53

^a^Sulfuric acid concentration given in% w/w of the dry-matter content,

^b^pH values of the solutions after pretreatment,

^c^Combined severity factor according to Overend *et al*. [47].

^d ^Decade logarithm of the combined severity factor; 1% w/w of the dry-matter content corresponds to a sulfuric acid concentration of 0.05 w/v%.

R'_0 _(R'_0 _= t × (10^-pH^) × exp × ((T-100)/14.75)),

and expressed as log R'_0 _according to Kabel *et al*. [16]). This factor is an indicator for the severity of a pretreatment, and takes into account the pretreatment temperature, time, acid concentration and final pH.

### Mass balance

**Figure 1:**
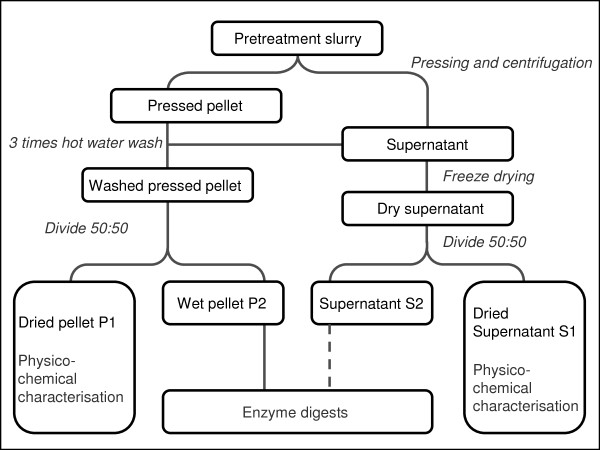
**Sample preparation of supernatants and pellets after pretreatment**.

The process of sample separation and mass-balance determination is summarized in Figure 1. Nylon pantyhose (15 denier) were used to filter the pretreated slurries. The remaining solids were wrapped in a double layer of cheesecloth, and pressed in a hydraulic press (IKA Werke GmbH & Co. KG, Staufen, Germany) at 100 bar until draining ceased. The pressed pellet was soaked three times with 500 ml distilled water at 60°C for 10 minutes, and pressed again. The supernatant was weighed and subsequently combined with the washes. The supernatant and pellets were both divided in two, and one half (S1 and P1) of each was freeze-dried for chemical characterization, and the other half (S2 and P2) was frozen for enzymatic digestion. The P1 fractions were weighed before and after freeze-drying, and the dry-matter content was determined as an indicator of the water-binding capacity after pretreatment. The S1 supernatants collected after pressing were spun in a centrifuge (12,320 × g) at 4°C for 20 minutes to remove cell debris. The clear S1 supernatants were freeze-dried, and the debris was added to the P1 pellet fraction. The S2 supernatants were concentrated to their original concentrations, and stored frozen (-20°C).

### Dry-matter content

Freeze-dried samples (20-40 mg) were weighed into aluminum cups (diameter 50 mm) and dried in an oven at 70°C for up to 3 days, until the weight was constant.

### Chemical characterization of pretreated SBP

After freeze-drying, the P1 pellets were weighed and ground (4 minutes at 100 Hz) in a swinging mill (Model M2200; Retsch GmbH, Haan, Germany) using 25 ml stainless steel containers with two stainless-steel metal balls (diameter 10 mm) per container.

The freeze-dried S1 supernatants were treated in the same way except for the supernatants S1 0-0, S1 170-0 and S1 170-1, which did not crystallize but instead formed a syrup. For these fractions, the syrup was dissolved in distilled water, and the volume was determined. An aliquot (1 ml) was freeze-dried and subsequently oven-dried to calculate the dry-matter content of the solutions. The different crystallization behavior did not affect the subsequent results.

### Enzyme digestion

#### Activity assays

Glycosidase activities in the experimental enzyme preparation C1-G1 were measured by determining the hydrolysis of *p*-nitrophenyl-β-d-glucopyranoside, *p*-nitrophenyl-β-d-xylopyranoside or *p*-nitrophenyl-β-l-arabinofuranoside at 37°C after 10 minutes of incubation. The reaction mixture consisted of 25 μl substrate solution (4 mg/ml) and 10 μl of the suitable enzyme concentration in 50 mmol/l sodium acetate buffer pH 5.0. The reaction was stopped by adding 40 μl of reaction mixture to 60 μl of 250 mmol/l TRIS-HCl buffer pH 8.8. The color formation was measured at 410 nm in a microtiter plate reader. One unit of activity was defined as 1 μmol of glucose, xylose or arabinose liberated per minute under the conditions specified. The molar extinction coefficient under these assay conditions was 0.00158/μmol/l/mm. Xylanase and cellulase activities were determined by measuring the amount of reducing sugars released from the hydrolysis of medium-viscosity wheat arabinoxylan (Megazyme International Ireland Limited, Bray, Wicklow, Ireland) and phosphoric acid-treated cellulose [17]. The reaction mixture consisted of 50 μl substrate solution (15 mg/ml) and 20 μl of the suitable enzyme concentration in 60 mmol/l sodium acetate buffer pH 5.0. The reaction mixtures were preheated at 37°C before the enzyme was added. After an incubation time of 10 minutes, 25 μl of the reaction mixture were used to determine the amount of reducing sugars using a *PAra*-Hydroxy Benzoic Acid Hydrazide (PAHBAH) assay [18].

The activities of polygalacturonase, rhamnogalacturonase, arabinanase and galactanase were determined by measuring the amount of reducing sugars released from the hydrolysis of poly-GA (Fluka, Sigma-Aldrich, Schnelldorf, Germany), linear arabinan (Megazyme) and potato galactan (Megazyme).The reaction mixture consisted of 180 μl substrate (5 mg/ml) in 50 mmol/l sodium acetate buffer pH 5.0, which was preheated to 37°C before 20 μl of the relevant enzyme concentration were added. The reaction mixture. After an incubation time of 20 minutes, 10 μl of the reaction mixture were used to determine the amount of reducing sugars using the PAHBAH assay [19]. Calibration curves of the different monosaccharides (50 to 750 μg/ml) were used for quantification.

#### Digestions of pretreated pulp

Pressed pulp P2 (5% w/w, 0.2% of the initial dry-matter) was digested with 10 μl of an experimental enzyme mixture (protein content 75 mg/ml, 1% w/w enzyme level) (C1-G1; Dyadic Netherlands, Wageningen, The Netherlands) in 100 mmol/l sodium acetate buffer pH 5.5 for 2, 5, 10, 24 or 48 hours. C1-G1 is an enzyme preparation of the fungus *Chrysosporium lucknowense *[20], which is rich in cellulase and xylanase activities, but also contains arabinanase and galactanase activities (Table 2). Ampicillin 100 μg/ml was added to prevent microbial growth. The reactions were carried out in 2 ml microcentrifuge tubes placed in an microcentrifuge tube shaker kept at 40°C and shaken at 800 rpm. Enzymes were inactivated by placing in a waterbath at 100°C for 15 minutes. The samples were spun in a centrifuge for 10 minutes at 20,000 × g and 20°C, and 200 μl of the supernatant was used for high-performance size exclusion chromatography (HPSEC) and high-performance anion exchange chromatography (HPAEC) analysis. The sugar composition of the remaining pellets was analyzed by gas chromatography with flame ionization detection (GC-FLD) after alditol acetate derivatization.

**Table 2:** Activity profile of enzyme mix C1-G1

Activity	U/mg protein
β-Glucosidase	0.198
β-Xylosidase	0.012
α-Arabinofuranosidase	0.035
Xylanase	1.322
Cellulase	1.400
Arabinanase	0.100
Galactanase	0.142
Polygalacturonase/pectate lyase	0.006

### Chromatography

#### Sugar composition

The neutral sugar composition was determined by analyzing the sugars as their alditol acetate derivatives using GC-FLD. A gas chromatograph (Thermo Focus; Thermo Scientific, Waltham, MA, USA) and autosampler (Focus AS 3000; Thermo Scientific) equipped with a column (15 m × 0.53 mm, 1 μm film; DB-225; J&W Scientific, Santa Clara, IL, USA) was used. The samples were hydrolyzed stepwise with 72% w/w sulfuric acid for 1 hour at 30°C, followed by 1 M sulfuric acid for 3 hours at 100°C. Subsequently, the free monosaccharides were reduced and derivatized to their corresponding alditol acetates [21]. The samples were dissolved in 0.4 ml acetone, and 1 μl was injected with a pre-injection dwell time of 1 second and a post-injection dwell time of 2 seconds. The sample was then eluted using the following temperature profile: 0 to 2 minutes at 180°C, 2 to 17 minutes at a linear gradient from 180 to 210°C, and 17 to 22 minutes at 210°C.

UA content was determined colorimetrically [22] with 3-phenylphenol using an automated analyzer (Skalar Analytical, Breda, The Netherlands). A GA standard curve (12.5 to 100.0 μg/ml) was used for quantification.

#### Organic-acid content

The content of organic acids, HMF, furfural and methanol was analyzed by high performance liquid chromatography with a (Ultimate 3000; Dionex Corp., Sunnyvale, CA, USA) equipped with an ion-exclusion column (300 mm × 7.8 mm; Aminex HPX-87H; Bio-Rad Laboratories, Hercules, CA, USA) in combination with a self-packed guard column (50 mm × 7.8 mm; AG 50W-X4 resin; Bio-Rad Laboratories). Samples were eluted with 5 mmol/l sulfuric acid at a flow rate of 0.6 ml/min at 30°C. The S1 supernatants were filtered through a 0.2 μm round filter (Whatman), and 20 μl samples used for injection. The samples were quantified by single-component standards of known concentrations (0.5 mg/ml). Elution was monitored by refractive index detection (Shodex RI 101; Showa Denko K.K., Kawasaki, Japan).

#### Monomer and oligomer analysis

The monosaccharide and oligosaccharide levels of the digests were analyzed by HPAEC with pulsed amperometric detection (PAD) (ITS-3000 HLPC; Dionex) equipped with an analytical column (2 mm × 250 mm, CarboPac PA1; Dionex) in combination with a guard column (2 mm × 50 mm, CarboPac PA1; Dionex) at a flow rate of 0.3 ml/min. Arabinose, rhamnose, galactose, glucose, xylose, mannose and GA monosaccharides in the range from 2 to 30 μg/ml were used for quantification. Oligomeric standards included arabinose oligomers (degree of polymerization (DP) 2 to 6, 10 μg/ml; Megazyme), GA oligomers (DP 2 and DP 3, 10 μg/ml), a potato galactan digest containing galactose oligomers (DP 2 to 5; 5 mg/ml potato galactan; Megazyme) partially digested with *Aspergillus niger *endogalactanase (purified in our laboratory) and cellodextrins (DP2 to 7; kind gift of Dr Vladimir Farkas, Institute of Chemistry, Slovak Academy of Sciences, Bratislava, Slovakia). The samples (10 μl; 50 to 100 μg/ml) were eluted in post column addition mode with the following elution profile: 0.0 to 30.0 minutes in water, 30.0 to 30.1 minutes in 0.0 to 0.1 mol/l NaOH, 30.1 to 32 in 0.1M NaOH, 32.0 to 62.5 minutes in 0.0 to 0.4 mol/l NaOAc in 0.1 mol/l NaOH, 62.5 to 67.0 minutes in 1.0 mol/l NaOAc dissolved in 0.1 mol/l NaOH, 67.0 to 75.0 minutes in 0.1 mol/l NaOH, and 75.1 to 90.0 minutes in water (equilibration). NaOH (0.5 mol/l, 0.1 ml/min) was added via a post column to allow PAD detection from 0.0 to 30.1 minutes and from 68.5 to 90.0 minutes.

#### Ferulic acid analysis

The amount of ester-linked ferulic acid was determined after alkaline hydrolysis and ethyl ether extraction as described previously [23] using reversed phase ultra-high-performance liquid chromatography (UHPLC) on a UHPLC system (Accela; Thermo Scientific) equipped with a column (2.1 mm × 150 mm, 1.9 μm particle size; Hypersyl GOLD; Thermo Scientific). Elution was monitored with the built-in polydiode array multi-wavelength detector set at 335 nm.

#### Molecular mass distribution

HPSEC was performed on a system (Ultimate 3000; Dionex) equipped with four columns (TSK-Gel superAW; Tosoh Bioscience, Tokyo, Japan) in series: a guard column (6 mm × 40 mm) and three separation columns (4000, 3000 and 2500; 6 mm × 150 mm). The samples (10 μl, 5 mg/ml) were eluted with 0.2 mol/l sodium nitrate at 40°C at a flow rate of 0.6 ml/min. Molecular masses were estimated with the help of molecular mass standards (Pullulan; Polymer Laboratories, Varian Inc., Palo Alto, CA, USA). Elution was monitored by refractive index detection (Shodex RI 101) and UV detection (internal detector) at four wavelengths (λ = 235 nm, 310 nm, 325 nm and 345 nm). The relative peak area distribution was calculated by the integration of the peak area in three time increments. The total peak area was calculated as the total peak area from 7.0 to 18.0 minutes without the negative peak (15.5 minutes) plus the peak area difference between the negative peak of a water blank and the negative peak of the supernatant sample.

#### Protein content

The protein content of the pretreated samples was determined by combustion (the Dumas method) with a nitrogen analyzer (FlashEA 1112 series; Thermo Scientific) in accordance with the manufacturer's instructions, using methionine as a standard and a nitrogen factor of 6.25.

## Results and Discussion

### Mass of pretreated SBP

**Figure 2:**
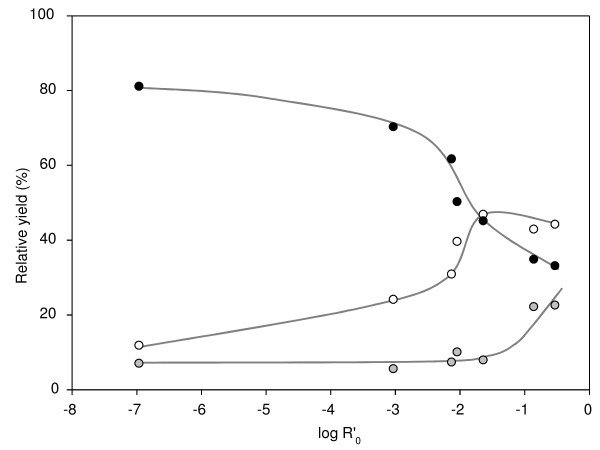
**Distribution of pretreated sugar-beet pulp over supernatant fractions, pellet fractions and decomposed material in relation to the combined severity factor (log R'0)**. Black dots = pellets, white dots = supernatants, grey dots = decomposed material. Lines indicate trends.

The effects of the different pretreatments on the solubilization of SBP were evaluated by weighing on a mass balance, taking into account the respective amounts of solubilized, residual and decomposed material. The combined severity factors (from the equation given in Methods) of the pretreatments are given in Table 1. The mass distribution of the samples after pretreatment was plotted against log R'_0 _(Figure 2). A log R'_0 _of around -3.0 to -1.5 was necessary to solubilize substantial parts of the beet pulp. With increasing severity of the pretreatment, an increasing amount of sample was lost, probably by chemical destruction of carbohydrates (for example, Maillard reaction) and formation of volatile compounds. Such formation of volatile compounds was seen during pretreatments with log R'_0 _≥-0.86 (samples 170-1 and 170-0), for which a temperature-independent pressure increase was observed after pretreatment (see Additional file 1, Figure S1). A strong color change after pretreatment and a caramel-like aroma indicated maillardation under the conditions used (data not shown).

Generally, more material was solubilized with increasing pretreatment severity, but a loss of mass was seen for all samples. These losses were mainly attributable to the mechanical loss of material during blending and sample handling. When this mechanical loss was averaged over the first five pretreatments, it was estimated to account for roughly 8% w/w. Pretreatments with log R'_0 _≥-0.86 (sample 140-1) led to major losses in mass, probably due to chemical decomposition, and no further increase in the level of soluble compounds in the supernatant (Figure 2). The pellet and supernatant curves were both sigmoidally shaped, which could imply that the range between solubilization and chemical degradation of the pulp is narrow, and hence requires good process control. If more severe treatments are required for effective disruption of the cellulose network, solubilized temperature-labile compounds should first be removed from the reactor to minimize their destruction. Such removal of soluble compounds could be carried out by using a flow-through reactor setting or an extruder.

### Sugar composition of released and residual carbohydrate material

**Figure 3:**
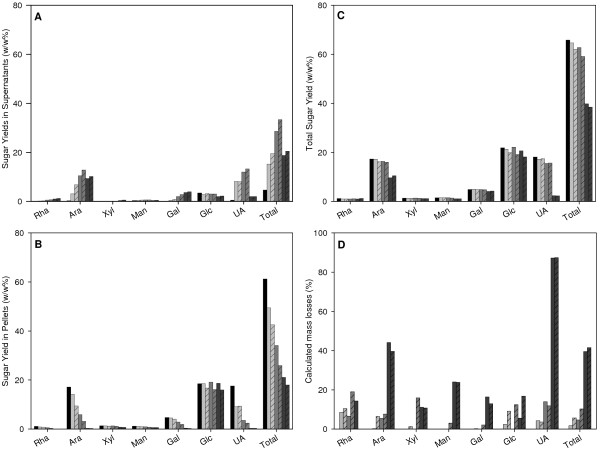
**Sugar yields of pretreated sugar-beet pulp fractions**. The data are expressed as percentage w/w based of starting material before pretreatment. Sugar yields of **(A) **the supernatant fractions, **(B) **the pellet fractions, **(C) **total pretreated material (sum of (A) + (B), **(D) **Calculated individual sugar losses during pretreatment. Black bars = untreated pulp, light-grey bars = 120°C fractions, grey bars = 140°C fractions, dark-grey bars = 170°C fractions, shaded bars = pretreatments with 1% w/w sulfuric acid (on a dry-matter basis), non-shaded bars = pretreatments in water.

The predominant sugars released were the pectin-associated sugars arabinose, galactose and GA, and there were also some traces of rhamnose, derived from the rhamnogalacturonan part of pectin (Figure 3A). In contrast to the increasing levels of solubilized pectin-derived sugars, around 3% w/w of the glucose component was released irrespective of the severity of the pretreatment. This released glucose was probably derived from the residual saccharose in the pressed pulp. When pretreated at 170°C (log R'_0 _≥-0.86), the arabinose and GA yields in the supernatant fraction decreased, whereas the levels of the others sugars remained constant or increased further. Decomposition levels of 40% and 90% were calculated for arabinose and GA, respectively, suggesting that arabinose and GA are both heat-labile (Figure 3D). Pectins have been shown to decarboxylate at high temperatures and high acid levels [24], leading to the formation of furfural [25] and/or arabinose [26]. Therefore, the apparent arabinose levels we detected might be a result of arabinose being lost during pretreatment and new arabinose chains being formed during the decomposition of GA.

High solubilization of arabinan and galacturonan from SBP after exposure to temperatures of 150 to 175°C has been reported previously [14,27]. Temperatures above 160°C led to decreasing recovery of GA, whereas arabinose recovery was stable up to 167°C [27], and most of the cellulose remained insoluble under the conditions used. Our data are in line with these observations.

We found that the sugar yields in the pellets decreased in line with the increasing severity of pretreatment (Figure 3B). Approximately 30% of the total sugars present in the starting material were left in the pellet fraction of sample 170-1. The absolute amounts of glucose remained almost constant, so that the relative glucose content of the pellet fractions increased up to 90% (moles) (see Additional file 2, Table s1). Even though cellulose was not solubilized during pretreatment, the solubilization of arabinan might also have had a positive effect on the enzymatic degradation of cellulose. It has been shown previously that arabinan can absorb to cellulose *in vitro *[28], thus removal of arabinan would be expected to lead to greater exposure of the cellulose surface to enzymes.

When the yields of the individual sugars present in the pellet and supernatant fractions were summed (Figure 3C), a decrease was seen for the pretreatments with log R'_0 _≥-0.86 (samples 170-0 and 170-1), which was similar to the yield of the total material after pretreatment (Figure 2).

### Sugar-degradation products formed during pretreatment

**Figure 4:**
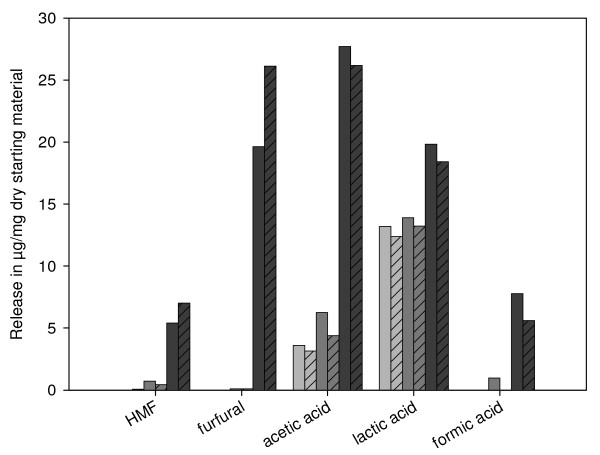
**Specific sugar-degradation products formed after pretreatment**. Light-grey bars = 120°C pretreatment, grey bars = 140°C pretreatment, dark-grey bars = 170°C pretreatments, shaded bars = pretreatments with 1% w/w sulfuric acid (on a dry = matter basis), non = shaded bars = pretreatments in water.

Besides lowering the yields, biomass pretreatment carries the risk of forming undesired degradation products that could negatively influence later use, such as in yeast fermentation for bioethanol production. We therefore investigated the samples to identify formation of formic acid, acetic acid, lactic acid, furfural and HMF (Figure 4). HMF, furfural and formic acid were only seen for pretreatments with log R'_0 _≥-0.86 (samples 170-0 and 170-1), which were both treated at 170°C. HMF formation in these samples may be linked to the thermal decomposition of fructose, which derives from the autohydrolysis of saccharose. It has been shown previously that fructose is more acid-labile and five times more reactive than glucose [29], probably due to a higher level of open-ring conformation at higher temperatures [30]. HMF formation for the samples 170-0 (0.55% w/w, 0.04 μmol) and 170-1 (0.71% w/w, 0.06 μmol) may be a result of the degradation of 33 to 50% of the saccharose-derived fructose (2% w/w, 0.12 μmol in SBP).

Furfural formation was linked to pentose and UA degradation [25,31], and only occurred in samples 170-0 (2.1% w/w, 0.2 μmol) and 170-1 (2.6% w/w, 0.25 μmol). This could explain 14 to 18% of the arabinose degradation (around 1.4 μmol in the pulp) or 20 to 25% of the UA degradation (around 1 μmol) we observed. Because 40% of the arabinose and 90% of the GA were decomposed (Figure 3D), it is likely that destruction of arabinose and GA also occurs via different mechanisms or yields different end-products.

For example, pectins can undergo β-elimination at high temperatures to form unsaturated GA and unsaturated GA oligomers [32]. A combination of β-elimination and decarboxylation, resulting in a pyranose ring structure of unsaturated 4,5-l-arabinopyranose and carbon dioxide, has also been proposed[33]. Carbon dioxide could be responsible for the temperature-independent pressure increase we saw during the 170°C pretreatments (see Additional file 1, Figure S1).

Formation of formic acid may be linked to further degradation of furfural and HMF at higher temperatures [31]. Furfural and HMF may also polymerize to form humins under acidic conditions and higher temperatures [34]; however, humin formation cannot explain the mass loss we found at log R'_0 _≥-0.86. In addition, it generally requires higher acid concentrations [34]. The presence of acetic and lactic acid in all our pretreated samples may also indicate contamination of the SBP starting material with acid-releasing bacteria. The release of acetic and lactic acid strongly increased at log R'_0 _≥-0.86. The acetic acid release (up to 2.5% w/w) can partly be explained by pectin deacetylation, because SBP contains around 1.6% w/w ester-linked acetic acid. In addition, acetic and lactic acid were also formed during the Maillard reaction.

The data from these experiments suggest that pretreatments at log R'_0 _≥-0.86 led to the enhanced destruction of arabinose and GA and to the production of toxic compounds that could negatively affect fermentation.

### Compressibility of pretreated pulp

The compressibility was studied as an indicator of the water-binding capacity (WBC) of the SBP. Water binding in plant cell walls of dicotyledons and non-graminaceous monocotyledons is usually attributable to pectin [35]. The pellets obtained after pretreatment had reduced levels of pectic arabinan and galacturonan (Figure 3B), but not all had reduced WBC (Table 3). The pellets of the 120°C and 140°C samples, by contrast, retained up to 207% more water than the untreated pulp. The 140°C pretreated samples had a water content 50% higher than the untreated pulp, whereas their pectin content was decreased by 70 to 80% (Figure 3B). The 170°C pretreated pellets were almost completely devoid of pectic sugars (Figure 3B), and after pressing, they retained only half the amount of water that the pressed untreated pulp retained. Hence, the swelling behavior of SBP is not only related to its pectin content, but also depends on other parameters, such as the cell-wall architecture.

**Table 3:** Water-binding capacity of pretreated samples

Sample1	**Bound water, g/g pellet**^**a**^	Change, %
0-0	2.4	0
120-0	5.5	133
120-1	7.2	207
140-0	3.3	42
140-1	3.6	54
170-0	1.4	-41
170-1	1.2	-47

^a^Pellets were pressed at 100 bar after pretreatment until drainage stopped.

Increased swelling and WBC have been described previously for pellets of alkali-extracted beet fibers [36,37], even though these fibers had strongly reduced levels of pectic arabinan and galacturonan. Analogously, it has been reported for the cell-wall material of wheat flour that enzymatic removal of arabinoxylan increased the WBC of the residual, cellulose-rich material [38]. Possible explanations for the increased water binding include the total polysaccharide content, charge, crystallinity of the cellulose, specific surface area, and hydrophobic components [37].

These results indicate that dry-matter contents higher than 5% may not be possible for pretreatment and enzymatic digestion of SBP, because it is not possible to pump or stir the pulp efficiently. Bioethanol or biogas production requires higher substrate concentrations to make the downstream processing (for example, distillation/rectification) economically feasible. To allow higher dry-matter concentrations, the WBC could be reduced either enzymatically by the addition of an endopolygalacturonase or thermophysically by a more severe pretreatment. However, there are drawbacks: enzymatic degradation would produce GA, which may be toxic to the yeast, and severe pretreatment would drastically lower the yield by causing biomass degradation and release of inhibiting compounds. A fed-batch setup could be a promising alternative to allow high dry-matter concentrations.

### Molecular mass distribution of solubilized material

**Figure 5:**
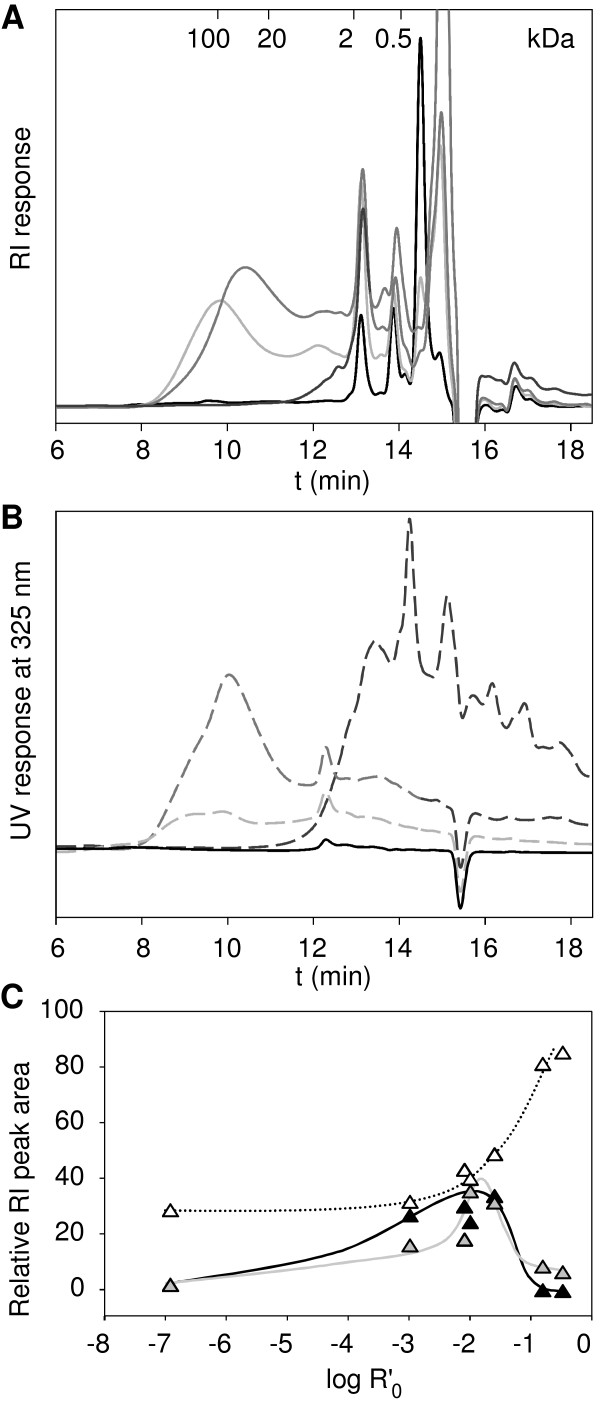
**Molecular mass distribution of solubilized material**. **(A,B) **High performance size exclusion chromatography (HPSEC) elution pattern of 0-0 (black), 120-1 (light grey), 140-1 (grey) and 170-1 (dark grey).**(A) **Solid lines = RI signal, **(B) **dashed lines = UV signal at 325 nm. **(C) **Logarithm of the combined severity factor (log R'_0_) vs. the relative RI peak area. Black triangles = peak area of the time interval 7.0 to 11.0 minutes, grey triangles = peak area of the time interval 11.0 to 13.0 minutes, white triangles = peak area of the time interval 13.0 to 15.2 minutes. Lines show trends.

The molecular mass distribution of the solubilized material was determined after pretreatment (Figure 5A). The 0-0 supernatant did not contain any polymeric material. The absence of water-soluble pectin in SBP may be explained by the prior industrial extraction of saccharose from sugar beet, which involved treatment with calcium hydroxide to precipitate soluble pectins. Three peaks in the small molecular mass region could represent residual saccharose and its hydrolysis products glucose and fructose, or some salts that were solubilized upon hot-water extraction.

The 120°C pretreatments both solubilized a polymeric fraction with an average molecular mass of approximately 100 kDa, based on the Pullulan molecular mass standards. Samples pretreated with and without sulfuric acid had the same molecular-mass distributions as the samples pretreated with water (data not shown). The average molecular mass of the solubilized material decreased with increasing temperatures from 50 kDa for the 140°C pretreatments to oligomeric material of ≤1 kDa at 170°C. Along with the solubilization of carbohydrates, an UV signal at 325 nm was detected for these populations, indicating the presence of phenols (Figure 5B).

Ferulic acid can be attached to pectic arabinan or galactan [39,40], and could explain the UV signal in the molecular mass range of ≥1 kDa. The UV signal in the molecular mass range of ≤1 kDa could indicate the presence of aromatic degradation products, such as furfural or HMF. The ferulic acid content of the supernatants was determined separately (Table 4) and compared with the HPSEC data (Figure 5B). The release of ferulic acid was important for two reasons. Ferulic acid esters present in arabinan and galactan can inhibit the complete degradation of biomass and lower the monosaccharide yields. Secondly, ferulic acid is a high value byproduct in itself, because it is a precursor of aromatic compounds, such as vanillin [41]. With increasing severity of the pretreatment, increasing amounts of ferulic acid were present in the solubilized fractions, which could explain the high UV absorption of the 140°C fractions at 10 minutes and the UV absorption of the 170°C samples in the oligomeric range at 13 to 15 minutes during the reaction.

**Table 4:** Ferulic acid (FA) yield in solubilized material after pretreatment

Sample	**FA**^**a **^**nmol/mg**	**FA**^**b**^**, %**
0-0	0	0
120-0	6	6
120-1	9	11
140-0	20	31
140-1	21	38
170-0	36	61
170-1	34	59

^a^Absolute amount of solubilized ester-linked FA.

^b^Relative amount of solubilized ester-linked FA.

An optimal pretreatment does not necessarily hydrolyze the insoluble material to monosaccharides and oligosaccharides, which are more prone to chemical destruction than are polymers. The molecular mass distributions of the supernatants indicated that the relative proportions of the high and average molecular mass material increased up to log R'_0 _= -1.65 (sample 140-1), whereas they strongly decreased for pretreatments with log R'_0 _≥-0.86 (Figure 5C). In turn, the amount of each compound eluting in the monomeric and oligomeric range increased with increasing severity. These results show, together with the decomposition (Figure 2; Figure 3D) that pretreatments with log R'_0 _≥-0.86 favored chemical destruction of sugars and were too severe when aiming for a maximum yield of fermentable sugars.

### Enzymatic degradation of pretreated SBP pellets

For bioethanol production, the aim is to ferment a wide range of monosaccharides. Besides hexose-fermenting yeast strains (mainly glucose and fructose but also galactose), several yeast strains that are able to ferment pentoses have been described in the literature [42]. The efficient enzymatic degradation of sugar-beet arabinan has already been reported [19]. To verify the influence of the pretreatment on enzymatic digestibility of cellulose, we degraded pretreated SBP pellets with an experimental enzyme mixture (C1-G1) that is rich in cellulases and xylanases (Table 2). This mixture has been designed for the degradation of wheat bran, but also has endogalactanase and endoarabinanase activities (Table 2). It is almost devoid of polygalacturonase/pectate lyase activity (Table 2).

Polygalacturonase deficiency could be beneficial for a subsequent yeast fermentation because high concentrations of weak acids have a negative effect on yeast viability, and could therefore lower ethanol production [10]. We were able to recover sugar-beet polygalacturonides after fermentation. These polygalacturonides can be used to produce pectin-derived oligosaccharides enzymatically. Pectin-derived oligosaccharides have been shown to have a number of beneficial biological effects, including prebiotic activity [43-45]. Alternatively, enzymatically released GA may be fermented to ethanol by bacteria [46].

We analyzed the supernatants of the enzyme digested pellets for their monomer sugar content, and calculated the yield for each individual sugar (Table 5). Glucose was the main monosaccharide released by C1-G1. Besides glucose, up to 4% of cellobiose was present after 48 hours, indicating insufficient β-glucosidase activity (data not shown). Glucose yields increased from 17% for the 0-0 pellet up to 91% for the 140-1 pellet, then dropped to 61 to 65% for the 170°C pellets. This pattern was confirmed by analysis of the sugar composition of the pellets after 48 hours of incubation with the enzyme (data not shown). A comparison of the glucose content of the starting material with that of the supernatants and pellets after enzymatic degradation confirmed that no glucose was destroyed. The reduced glucose release after the 170°C pretreatments could indicate enzyme inhibition, or a structural change in the cell-wall complex that made cellulose less accessible.

**Table 5:** Yields of individual monosaccharides ending up in the supernatant after enzyme incubation for 48 hours of pretreated pellets with C1-G1 enzyme mixture (1% w/w)^a^

Sample	Ara	Gal	Glc	Xyl	Man	**Total**^**b**^
0-0	6	3	17	6	3	9
120-0	12	4	75	14	20	36
120-1	29	4	83	21	23	52
140-0	24	3	86	21	23	58
140-1	24	2	91	18	26	62
170-0	0	0	61	19	9	54
170-1	0	0	65	22	9	59

^a^Values in % of total amount of individual sugar present in the pellets.

^b^Yield of all monosaccharides (% of total neutral sugar present in the pellets.

**Figure 6:**
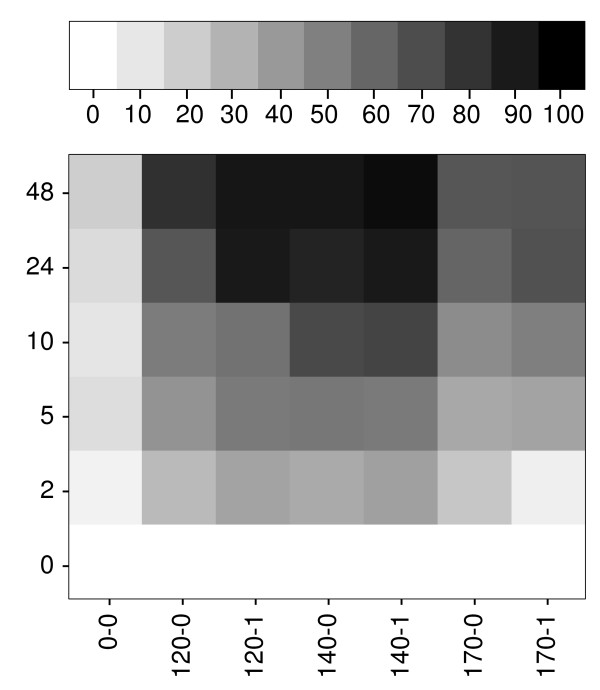
**Enzymatic release of glucose and cellobiose from pretreated sugar-beet pulp pellets**. Values were transformed into a heat map ranging from white (0% solubilization) to black (100% solubilization). Vertical labels show incubation times (hours), and samples are shown horizontally from left to right in order of increasing severity of pretreatment.

There was no GA release and only a small release of galactose seen. Some arabinose monosaccharides were released, reaching a yield of up to 29% based on the arabinose originally present in the pellet fraction. Monomer yields of arabinose and galactose could be further increased by the addition of arabinofuranosidases and galactosidases, respectively, because arabinose oligomers up to DP 6 and galactobiose were detected by HPAEC. Taken together, our data indicate that C1-G1 was able degrade up to 62% of all neutral sugars present in the pretreated pellets to monomers, including 91% of glucose originating from cellulose. The time-dependent enzymatic hydrolysis of cellulose from pretreated pellets was also studied (Figure 6). In general, cellulose hydrolysis was faster for the samples that were pretreated with sulfuric acid. For samples 120-1 and 140-1, the endpoint was almost reached after 24 hours, whereas 120-0 and 140-0 required 48 hours for maximal hydrolysis (Figure 6). Ultimately, more sugars were hydrolyzed with pretreatment 140-0 and subsequent enzyme digestion, because slightly higher losses occur at 140-1 (Figure 3D). Therefore, pretreatment 140-0 was the optimal pretreatment for the release of glucose.

### Total solubilization after pretreatment and enzymatic digestion

**Figure 7:**
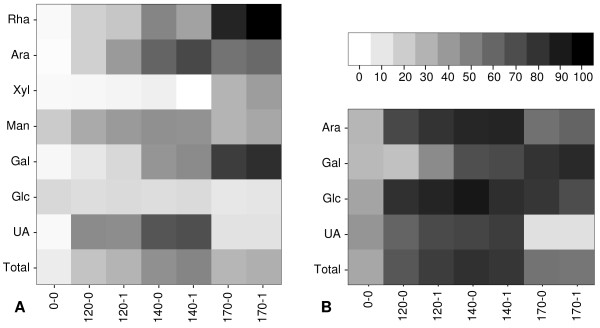
**Solubilization of individual sugars after pretreatment and subsequent enzymatic digestion**. Values were transformed into a heat map ranging from white (0% solubilization) to black (100% solubilization). **(A) **Solubilization of individual sugars after pretreatment (percentage compared with starting material). **(B) **Total yields of the main sugars that were solubilized after pretreatment and 24 hours of enzyme digestion. Values are expressed as % w/w of the sugar content of sugar-beet pulp before pretreatment. Ara = arabinose, Rha = rhamnose, Xyl = xylose, Man = mannose, Gal = galactose, Glc = glucose, UA = UAs, Total = total recovery of all sugars. Samples are shown horizontally from left to right in order of increasing severity of pretreatment.

Upon enzyme digestion of the pretreated pellets, the pellets of all pretreated samples were enriched in protein content from 8% w/w up to 25% w/w, indicating that the main part of the proteins remained insoluble during pretreatment, whereas their sugar content strongly decreased (data not shown). The influence of the pretreatment and enzymatic digestion on the solubility of each individual sugar was analyzed for all samples (Figure 7; see Additional file 3, Table s2). After pretreatment, the minor constituents rhamnose and galactose were effectively solubilized at 170°C, whereas the major constituents arabinose and UA (mainly GA) gave only a 60% yield at 140°C and were destroyed at higher temperatures (Figure 7A).

The total carbohydrate solubilization was calculated as the sum of all material solubilized after pretreatment plus the material solubilized from the pellet after enzymatic digestion (Figure 7B). The solubilization was inversely correlated with increasing severity, and ranged from 34% for the 0-0 digest to 80% for the 140-0 digest. The pectin-associated sugars arabinose, galactose and GA had 85, 70 and 75% solubilization, respectively, in sample 140-1. Mild pretreatments up to log R'_0 _= -1.65 generally enhanced the enzymatic degradation of cellulose from SBP (Figure 7B). C1-G1 also released material of high molecular mass from the pellets of samples 0-0, 120-0 and 120-1 (determined by HPSEC analysis, data not shown). It is likely that the solubilized material was pectin, because after enzyme incubation for 48 hours, the pellets had a strong reduction in their UA content (data not shown). Pectin can be solubilized either by the degradation of the cellulose network or by the removal of arabinan sidechains linking it to cellulose [28].

## Conclusions

We found that the combination of a mild hydrothermal pretreatment at 140°C with subsequent enzymatic digestion allowed fast and efficient hydrolysis of SBP. More than 90% of all cellulose could be hydrolyzed within 24 hours, using lower enzyme levels than those reported in earlier studies. In contrast to the chemical pretreatments described in literature, mild pretreatments up to 140°C did not result in a loss of biomass or in production of any compounds toxic to yeast. If the pretreatment process were to be implemented in a sugar factory, the pulp would already be at 70°C after saccharose extraction, and the exhaust heat of the lime ovens could be used in part for the heating of the SBP up to 140°C. The mild pretreatments did not lower the WBC of beet pulp and are therefore unsuitable for increasing the dry-matter content of SBP. Hence, to reduce transportation, pretreated SBP should be fermented in the vicinity of the sugar factory. A fed-batch reactor setting could allow a reasonably high ethanol concentration.

## Competing interests

The author declares that they have no competing interests.

## Authors' contributions

SK and HAS designed and coordinated the study. SK carried out the experiments and prepared the manuscript. HAS and HG contributed to the manuscript preparation. All authors read and approved the final version of the manuscript.

## Supplementary Material

Additional file 1 **Figure S1. Pressure and temperature curves of a 170°C pretreatment. **Black line = temperature (°C), grey line = pressure (bar). Click here for file

Additional file 2 Table S1: Molar sugar composition of pretreated samples. Click here for file

Additional file 3 **Table S2: Volumetric concentrations of the main sugars present in sugar-beet pulp obtained after pretreatment and 24 hours of enzyme digestion. **^a^Volumetric concentrations of arabinose (Ara), galactose (Gal), glucose (Glc) and uronic acid s (UA) present in the supernatant fraction (g/l). ^b^Amount of sugars (g) present in 50 g starting material. Click here for file

## References

[B1] McCready RM Polysaccharides of sugar beet pulp. A review of their chemistry J Am Soc Sugar Beet 1966 14 260 270 10.5274/jsbr.14.3.260

[B2] Kelly P Sugar beet pulp - A review Anim Feed Sci Tech 1983 8 1 18 10.1016/0377-8401(83)90038-X

[B3] Voragen AGJ Oosterveld A Schols HA Beldman G Pectic substances from sugar beet pulp: extraction and fractionation, structural features, functional properties and enzymic modification Transformation and modification of carbohydrates, Proc of 4th Int Workshop on carbohydrates as organic raw materials 1997 Vienna, Austria 29

[B4] Bettiga M Bengtsson O Hahn-Hägerdal B Gorwa-Grauslund MF Arabinose and xylose fermentation by recombinant *Saccharomyces cerevisiae *expressing a fungal pentose utilization pathway Microb Cell Fact 2009 8 10.1186/1475-2859-8-40 PMC2720912 19630951

[B5] Alvira P Tomás-Pejó E Ballesteros M Negro MJ Pretreatment technologies for an efficient bioethanol production process based on enzymatic hydrolysis: a review Bioresource Technol 2010 101 4851 4861 10.1016/j.biortech.2009.11.093 20042329

[B6] Abatzoglou N Chornet E Belkacemi K Overend RP Phenomenological kinetics of complex systems: the development of a generalized severity parameter and its application to lignocellulosics fractionation Chem Eng Sci 1992 47 1109 1122 10.1016/0009-2509(92)80235-5

[B7] Chum HL Johnson DK Black SK Overend RP Pretreatment-catalyst effects and the combined severity parameter Appl Biochem Biotech 1990 24-25 1 14 10.1007/BF02920229

[B8] Palmqvist E Hahn-Hägerdal B Fermentation of lignocellulosic hydrolysates. II: Inhibitors and mechanisms of inhibition Bioresource Technol 2000 74 25 33 10.1016/S0960-8524(99)00161-3

[B9] Banerjee N Bhatnagar R Viswanathan L Inhibition of glycolysis by furfural in *Saccharomyces cerevisiae* Eur J Appl Microbiol 1981 11 226 228 10.1007/BF00505872

[B10] Rottenberg H Fleischer S Packer L The measurement of membrane potential and [Delta]pH in cells, organelles, and vesicles Method Enzymol 1979 55 547 569 10.1016/0076-6879(79)55066-6 37402

[B11] Palmqvist E Hahn-Hägerdal B Fermentation of lignocellulosic hydrolysates. I: Inhibition and detoxification Bioresource Technol 2000 74 17 24 10.1016/S0960-8524(99)00160-1

[B12] Beldman G Rombouts FM Voragen AGJ Pilnik W Application of cellulase and pectinase from fungal origin for the liquefaction and saccharification of biomass Enzyme Microb Technol 1984 6 503 507 10.1016/0141-0229(84)90004-8

[B13] Chamy R Illanes A Aroca G Nunez L Acid hydrolysis of sugar beet pulp as pretreatment for fermentation Bioresource Technol 1994 50 149 152 10.1016/0960-8524(94)90067-1

[B14] Martínez M Gullón B Schols HA Alonso JL Parajó JC Assessment of the production of oligomeric compounds from sugar beet pulp Ind Eng Chem Res 2009 48 4681 4687 10.1021/ie8017753

[B15] Micard V Renard CMGC Thibault JF Influence of pretreatments on enzymic degradation of a cellulose-rich residue from sugar-beet pulp Lebensm Wiss Technol 1997 30 284 291 10.1006/fstl.1996.0182

[B16] Kabel MA Bos G Zeevalking J Voragen AGJ Schols HA Effect of pretreatment severity on xylan solubility and enzymatic breakdown of the remaining cellulose from wheat straw Bioresource Technol 2007 98 2034 2042 10.1016/j.biortech.2006.08.006 17029957

[B17] Walseth CS Occurrence of cellulase in enzyme preparation from microorganism TAPPI 1952 35 228 233

[B18] Lever M A new reaction for colorimetric determination of carbohydrates Anal Biochem 1972 47 273 279 10.1016/0003-2697(72)90301-6 5031119

[B19] Kühnel S Hinz SWA Pouvreau L Visser J Schols HA Gruppen H *Chrysosporium lucknowense *arabinohydrolases efficiently degrade sugar beet arabinan Bioresource Technol 2010 101 8300 8307 10.1016/j.biortech.2010.05.070 20566287

[B20] Hinz SWA Pouvreau L Joosten R Bartels J Jonathan MC Wery J Schols HA Hemicellulase production in *Chrysosporium lucknowense *C1 J Cereal Sci 2009 50 318 323 10.1016/j.jcs.2009.07.005

[B21] Englyst HN Cummings JH Simplified method for the measurement of total non-starch polysaccharides by gas-liquid chromatography of constituent sugars as alditol acetates Analyst 1984 109 937 942 10.1039/an9840900937 6283946

[B22] Ahmed ERA Labavitch JM A simplified method for accurate determination of cell wall uronide content J Food Biochem 1978 1 361 365 10.1111/j.1745-4514.1978.tb00193.x

[B23] Appeldoorn MM Kabel MA van Eylen D Gruppen H Schols HA Characterisation of oligomeric structures from corn fiber resistant towards pretreatment and simultaneous saccharification and fermentation J Agric Food Chem 2010 58 11294 11301 10.1021/jf102849x 20942461

[B24] Conrad CM Decarboxylation studies on pectins and calcium pectates J Am Chem Soc 1931 53 1999 2003 10.1021/ja01356a059

[B25] Dunlop AP Furfural formation and behavior Ind Eng Chem 1948 40 204 209 10.1021/ie50458a006

[B26] Zweifel G Deuel H Über die Decarboxylierung von Galakturonsäure mit Schwermetallionen Helv Chim Acta 1956 39 662 667 10.1002/hlca.19560390303

[B27] Martínez M Gullón B Yáñez R Alonso JL Parajó JC Kinetic assessment on the autohydrolysis of pectin-rich by-products Chem Eng J 2010 162 480 486 10.1016/j.cej.2010.05.048

[B28] Zykwinska AW Ralet MC Garnier CD Thibault JF Evidence for in vitro binding of pectin side chains to cellulose Plant Physiol 2005 139 397 407 10.1104/pp.105.065912 16126855 PMC1203388

[B29] Shallenberger RS Mattick LR Relative stability of glucose and fructose at different acid pH Food Chem 1983 12 159 165 10.1016/0308-8146(83)90002-X

[B30] van Dam HE Kieboom APG van Bekkum H The conversion of fructose and glucose in acidic media: formation of hydroxymethylfurfural Starch 1986 38 95 101 10.1002/star.19860380308

[B31] Ulbricht RJ Northup SJ Thomas JA A review of 5-hydroxymethylfurfural (HMF) in parenteral solutions Fund Appl Toxicol 1984 4 843 853 10.1016/0272-0590(84)90106-4 6391997

[B32] Voragen AGJ Schols HA Pilnik W Non-enzymatic browning of oligogalacturonides in apple juice models Z Lebensm Unters Forsch A 1988 187 315 320 10.1007/BF01454420

[B33] Einhorn-Stoll U Kunzek H The influence of the storage conditions heat and humidity on conformation, state transitions and degradation behaviour of dried pectins Food Hydrocolloid 2009 23 856 866 10.1016/j.foodhyd.2008.05.001

[B34] Hurd CD Isenhour LL Pentose reactions. I. Furfural formation J Am Chem Soc 1932 54 317 330 10.1021/ja01340a048

[B35] Reiter WD Biosynthesis and properties of the plant cell wall Curr Opin Plant Biol 2002 5 536 542 10.1016/S1369-5266(02)00306-0 12393017

[B36] Bertin C Rouau X Thibault JF Structure and properties of sugar beet fibres J Sci Food Agric 1988 44 15 29 10.1002/jsfa.2740440104

[B37] Rouau X Bertin C Thibault JF Characterization and enzymic degradation of sugar beet fibres Food Hydrocolloid 1987 1 439 443 10.1016/S0268-005X(87)80037-1

[B38] Gruppen H Kormelink FJM Voragen AGJ Enzymic degradation of water-unextractable cell wall material and arabinoxylans from wheat flour J Cer Sci 1993 18 129 143 10.1006/jcrs.1993.1041

[B39] Guillon F Thibault JF Rombouts FM Voragen AGJ Pilnik W Enzymic hydrolysis of the "hairy" fragments of sugar-beet pectins Carbohyd Res 1989 190 97 108 10.1016/0008-6215(89)84150-3

[B40] Rombouts FM Thibault JF Enzymic and chemical degradation and the fine structure of pectins from sugar-beet pulp Carbohyd Res 1986 154 189 203 10.1016/S0008-6215(00)90032-6

[B41] Mathew S Abraham TE Ferulic acid: An antioxidant found naturally in plant cell walls and feruloyl esterases involved in its release and their applications Crit Rev Biotechnol 2004 24 59 83 10.1080/07388550490491467 15493526

[B42] van Maris AJA Efficient ethanol production from arabinose Ind Bioproc 2007 29 9 10

[B43] Gullón P Gullón B Moure A Alonso JL Domínguez H Parajó JC Manufacture of Prebiotics from Biomass Sources Prebiotics and Probiotics Science and Technology 2009 535 589

[B44] Hotchkiss AT Olano-Martin E Grace WE Gibson GR Rastall RA Eggleston G, Côté GL Pectic oligosaccharides as prebiotics 2003 849 American Chemical Society, Washington, D. C., USA 54 62

[B45] Mandalari G Nueno Palop C Tuohy K Gibson GR Bennett RN Waldron KW Bisignano G Narbad A Faulds CB In vitro evaluation of the prebiotic activity of a pectic oligosaccharide-rich extract enzymatically derived from bergamot peel Appl Microbiol Biot 2007 73 1173 1179 10.1007/s00253-006-0561-9 17021882

[B46] Doran JB Cripe J Sutton M Foster B Fermentations of pectin-rich biomass with recombinant bacteria to produce fuel ethanol Appl Biochem Biotechn 2000 84--86 141 152 10.1385/ABAB:84-86:1-9:141 10849785

[B47] Overend RP Chornet E Gascoigne JA Fractionation of lignocellulosics by steam-aqueous pretreatments [and discussion] Phil Trans R Soc Lond A 1987 321 523 536 10.1098/rsta.1987.0029

