# Supplementary figures and images for: Aiming for the complete utilization of sugar-beet pulp: Examination of the effects of mild acid and hydrothermal pretreatment followed by enzymatic digestion
Source: Biotechnol Biofuels. 2011 May 31;4:14. doi: 10.1186/1754-6834-4-14 (PMC3130651; doi:10.1186/1754-6834-4-14)

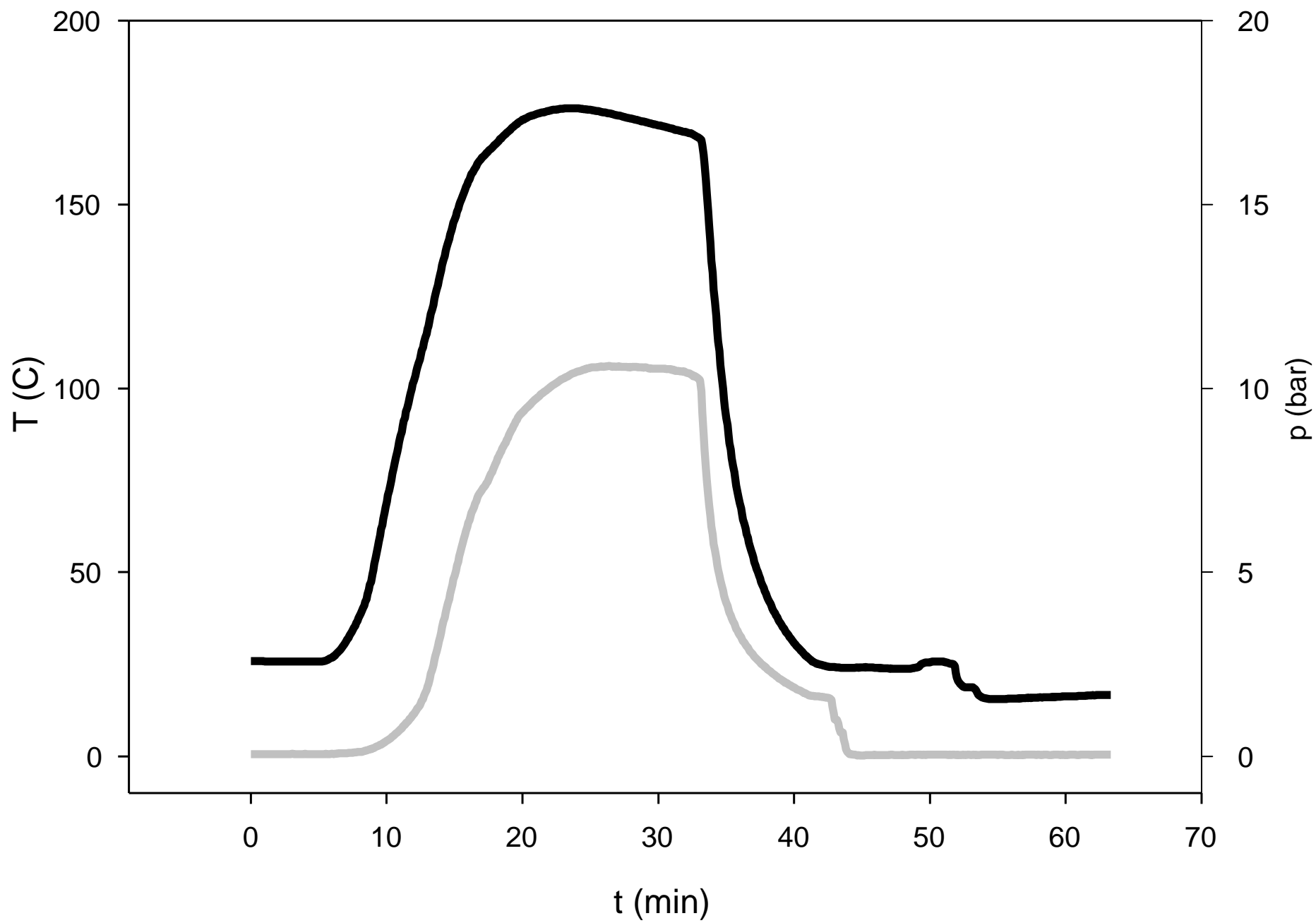

Supplement: Additional file 1 — Figure S1. Pressure and temperature curves of a 170°C pretreatment. Black line = temperature (°C), grey line = pressure (bar). [file 1754-6834-4-14-S1.PDF]
