# Supplementary material for: Aiming for the complete utilization of sugar-beet pulp: Examination of the effects of mild acid and hydrothermal pretreatment followed by enzymatic digestion
Source: Biotechnol Biofuels. 2011 May 31;4:14. doi: 10.1186/1754-6834-4-14 (PMC3130651; doi:10.1186/1754-6834-4-14)
Supplement: Additional file 2 — Table S1: Molar sugar composition of pretreated samples. [file 1754-6834-4-14-S2.DOC]

## Additional ﬁle 2 – Table s1: Molar sugar composition of pretreated samples.

|  | Rha | Ara | Xyl | Man | Gal | Glc | Uronic acids | Total sugar w/w% |
| --- | --- | --- | --- | --- | --- | --- | --- | --- |
| S-Bl | 1 | 6 | 1 | 6 | 3 | 73 | 10 | 39 |
| S-120-0 | 1 | 25 | 0 | 3 | 3 | 18 | 49 | 63 |
| S-120-1 | 1 | 40 | 0 | 3 | 4 | 15 | 63 | 63 |
| S-140-0 | 2 | 43 | 0 | 2 | 7 | 10 | 37 | 72 |
| S-140-1 | 2 | 45 | 0 | 2 | 8 | 8 | 35 | 70 |
| S-170-0 | 5 | 55 | 2 | 2 | 17 | 9 | 9 | 44 |
| S-170-1 | 6 | 55 | 3 | 2 | 17 | 10 | 8 | 46 |
| P-Bl | 2 | 33 | 2 | 2 | 7 | 29 | 35 | 75 |
| P-120-0 | 2 | 33 | 3 | 2 | 9 | 35 | 16 | 70 |
| P-120-1 | 2 | 26 | 3 | 2 | 9 | 38 | 19 | 69 |
| P-140-0 | 2 | 20 | 4 | 3 | 8 | 54 | 9 | 68 |
| P-140-1 | 1 | 15 | 5 | 3 | 7 | 60 | 8 | 62 |
| P-170-0 | 0 | 2 | 4 | 3 | 2 | 88 | 1 | 60 |
| P-170-1 | 0 | 2 | 4 | 3 | 1 | 88 | 1 | 54 |
