# Supplementary material for: Aiming for the complete utilization of sugar-beet pulp: Examination of the effects of mild acid and hydrothermal pretreatment followed by enzymatic digestion
Source: Biotechnol Biofuels. 2011 May 31;4:14. doi: 10.1186/1754-6834-4-14 (PMC3130651; doi:10.1186/1754-6834-4-14)
Supplement: Additional file 3 — Table S2: Volumetric concentrations of the main sugars present in sugar-beet pulp obtained after pretreatment and 24 hours of enzyme digestion. aVolumetric concentrations of arabinose (Ara), galactose (Gal), glucose (Glc) and uronic acid s (UA) present in the supernatant fraction (g/l). bAmount of sugars (g) present in 50 g starting material. [file 1754-6834-4-14-S3.DOC]

## Additional ﬁle 3 – Table s2: Volumetric concentrations of the main sugars present in sugar beet pulp obtained after pretreatment and 24 h enzyme digestion.

^1^Volumetric concentrations of arabinose (Ara), galactose (Gal), glucose (Glc) and uronic acids (UA) present in the supernatant fraction (g/l), ^2^amount of sugars (g) present in 50 g starting material.

| Initial^2^ | Ara (9.0) | Gal (2.5) | Glc (11.0) | UA (9.0) | Total^1^ (31.5) |
| --- | --- | --- | --- | --- | --- |
| 0–0 | 2.5 | 0.7 | 3.9 | 3.7 | 10.7 |
| 120–0 | 6.4 | 0.6 | 8.8 | 5.4 | 21.2 |
| 120–1 | 7.1 | 1.1 | 9.4 | 6.3 | 23.9 |
| 140–0 | 7.6 | 1.7 | 9.9 | 6.5 | 25.6 |
| 140–1 | 7.7 | 1.8 | 8.9 | 6.8 | 25.1 |
| 170–0 | 5.0 | 2.0 | 8.6 | 1.1 | 16.6 |
| 170–1 | 5.4 | 2.1 | 7.6 | 1.1 | 16.1 |
